# Supplementary material for: Insights into the mechanism of coreactant electrochemiluminescence facilitating enhanced bioanalytical performance
Source: Nat Commun. 2020 May 29;11:2668. doi: 10.1038/s41467-020-16476-2 (PMC7260178; doi:10.1038/s41467-020-16476-2)
Supplement: Supplementary file 1 — Supplementary Information [file 41467_2020_16476_MOESM1_ESM.pdf]

Supplementary Information

Insights into the mechanism of coreactant electrochemiluminescence facilitating enhanced bioanalytical performance

Zanut et al.

Supplementary Figures

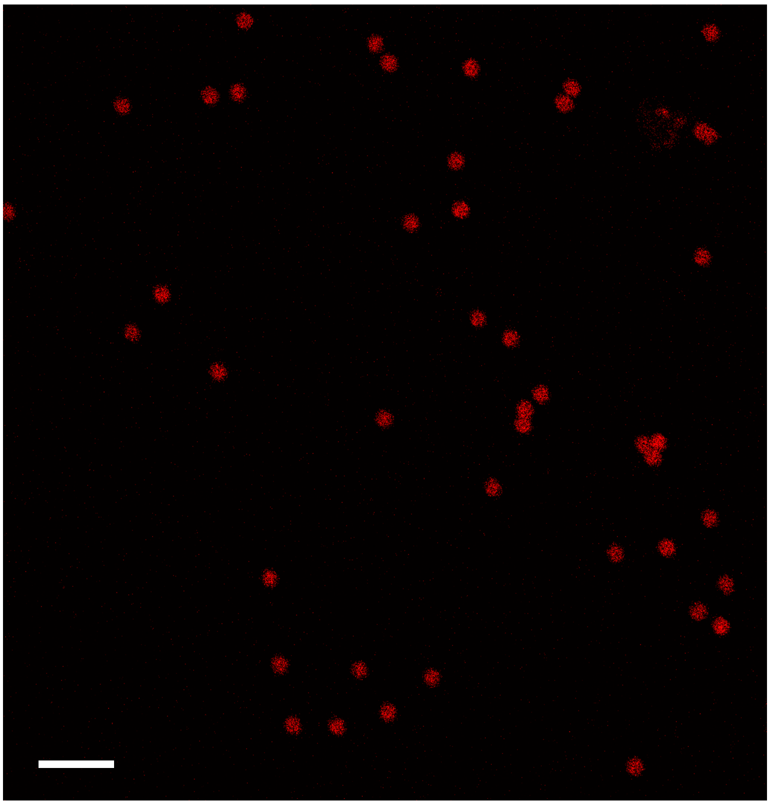

Supplementary Figure 1. *Ru@beads* Confocal image. Confocal Image of 2.8 µm beads, labelled with $[Ru(bpy)_3]^{2+}$ complex, deposited on the electrode surface. Magnification 20X. Scale bar, 20 µm.

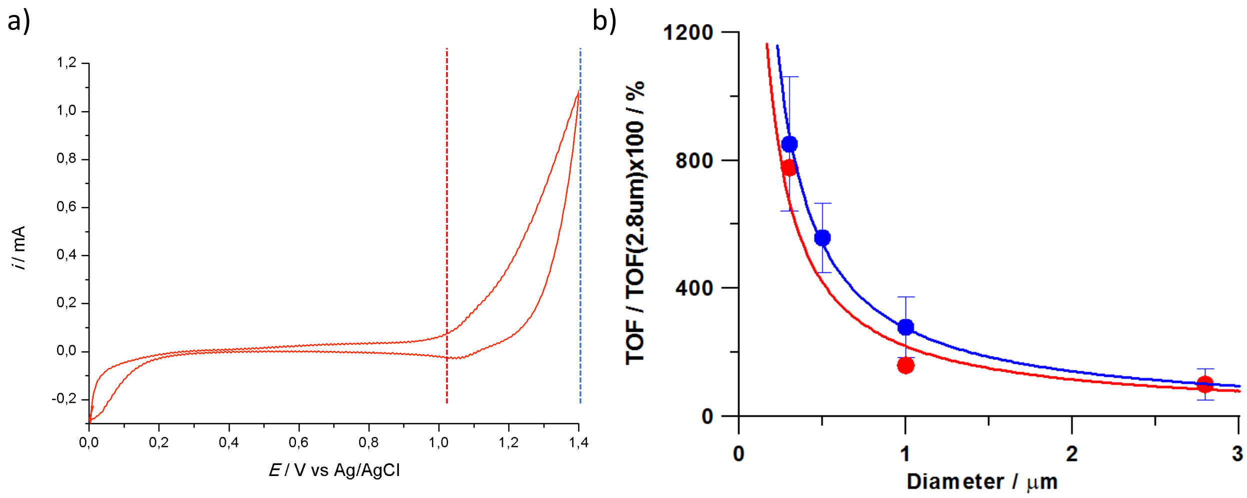

Supplementary Figure 2. Effect of the potential on the surface generation beads emission. a) Cyclic voltammetry of the of 0.5 mM $Ru(bpy)_3^{2+}$/PB solution showing the potential used for the surface generation–bead emission experiment. **b)** Surface generation–bead emission experiment performed at 1 V (red line) and at 1.4 V (blue line), respectively, before and after the potential for the $Ru(bpy)_3^{2+}$ oxidation. Error bar shows the standard deviation (n=10).

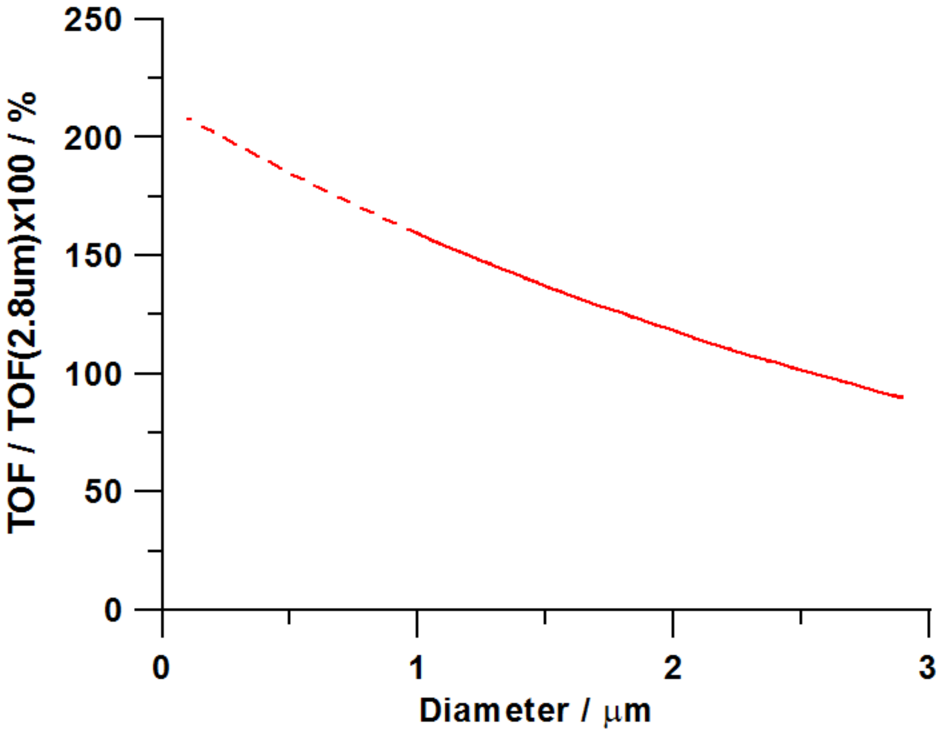

Supplementary Figure 3. State-of-art of ECL intensity as function of substrate distance. Exponential increase in ECL intensity as a function of the substrate distance data adapted from reference 2.

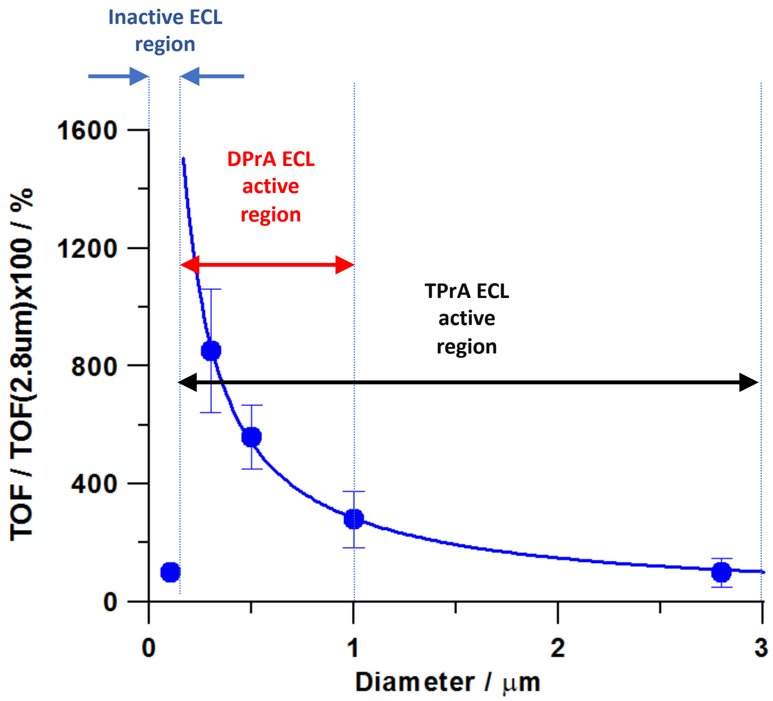

Supplementary Figure 4. Spatial map of the ECL emission. ECL intensity as a function of bead dimension (blue curve and dots) highlighting the mechanism that is active at the various distances. Error bar shows the standard deviation (n=10).

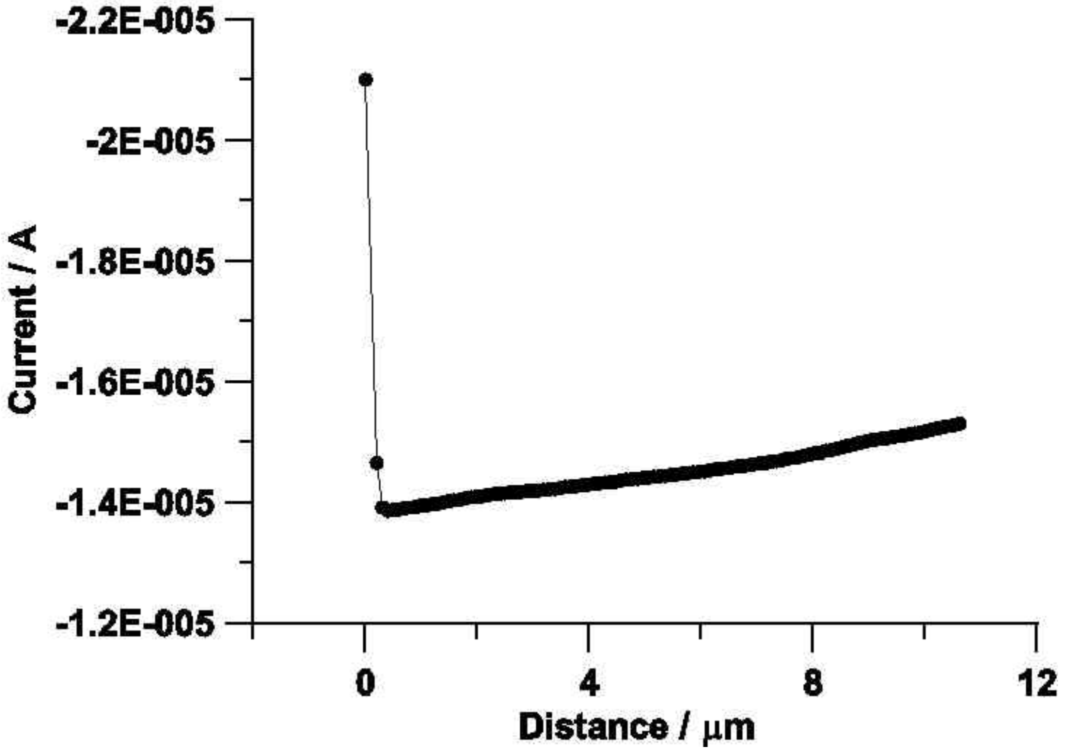

Supplementary Figure 5. Tip approach curve. Distance-current calibration for the oxidation of TPrA by Pt hemispherical electrode approaching on the ITO modified surface. Constant potential applied of 1.2 V vs Ag/AgCl, TPrA 180 mM in PB 0.2 M (pH 6.9).

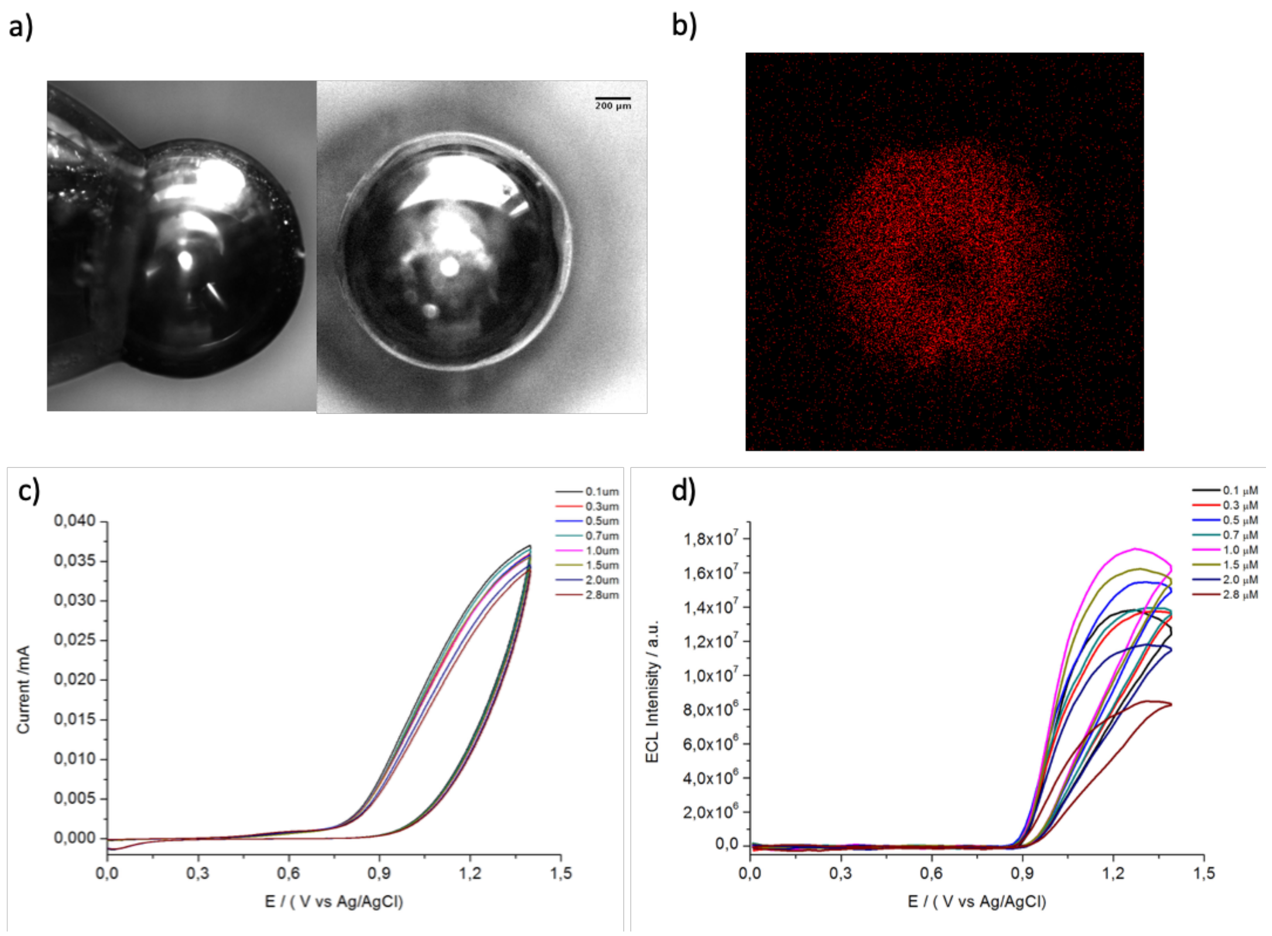

Supplementary Figure 6. Hemispherical large electrode characterization. Images of **a)** lateral and front optical view and **b)** ECL emission generated at 1 µm from the ITO surface modified with Ru complex at 1.4 V, Magnification 4x. Cyclic voltammograms **c)** currents and **d)** corresponding ECL signals at different distance (0.1 – 2.8 µm) from the ITO modified surface applying a potential range of 0-1.4 V, scan rate 100 mV s^{-1}. All measurements were obtained in 0.2 M PB with TPrA 180 mM (pH 6.9), potential vs Ag/AgCl (3 M KCl). Scale bar, 200 µm.

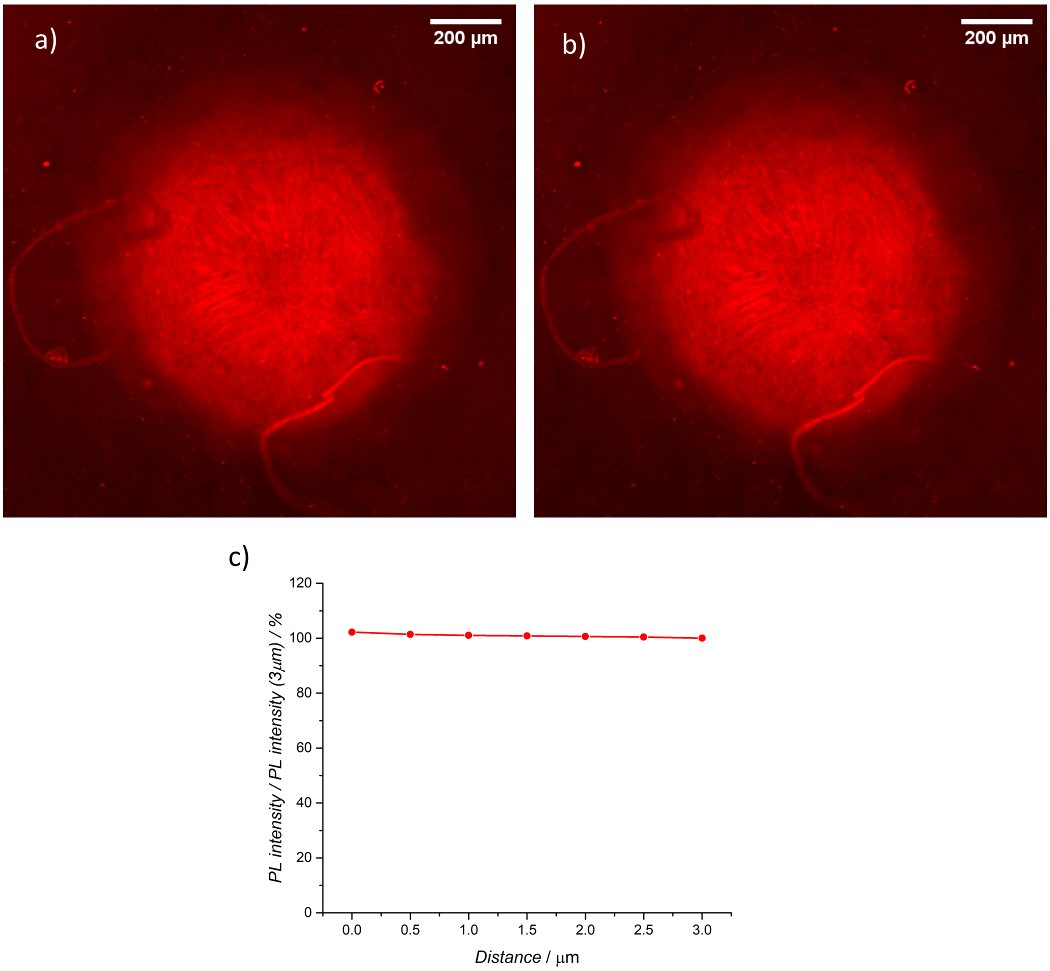

Supplementary Figure 7. Reflection of the electrode tip. Images of fluorescence intensity acquired for a rhodamine-functionalized glass slide under the large Pt hemispherical electrode (diameter = 1.5 mm). Distance of **a)** 1 µm and **b)** 3 µm from the tip to the glass. **C)** Integrated intensity as a function of the tip–glass distance. Magnification 4x; filter, Texas Red®. Scale bar, 200 µm.

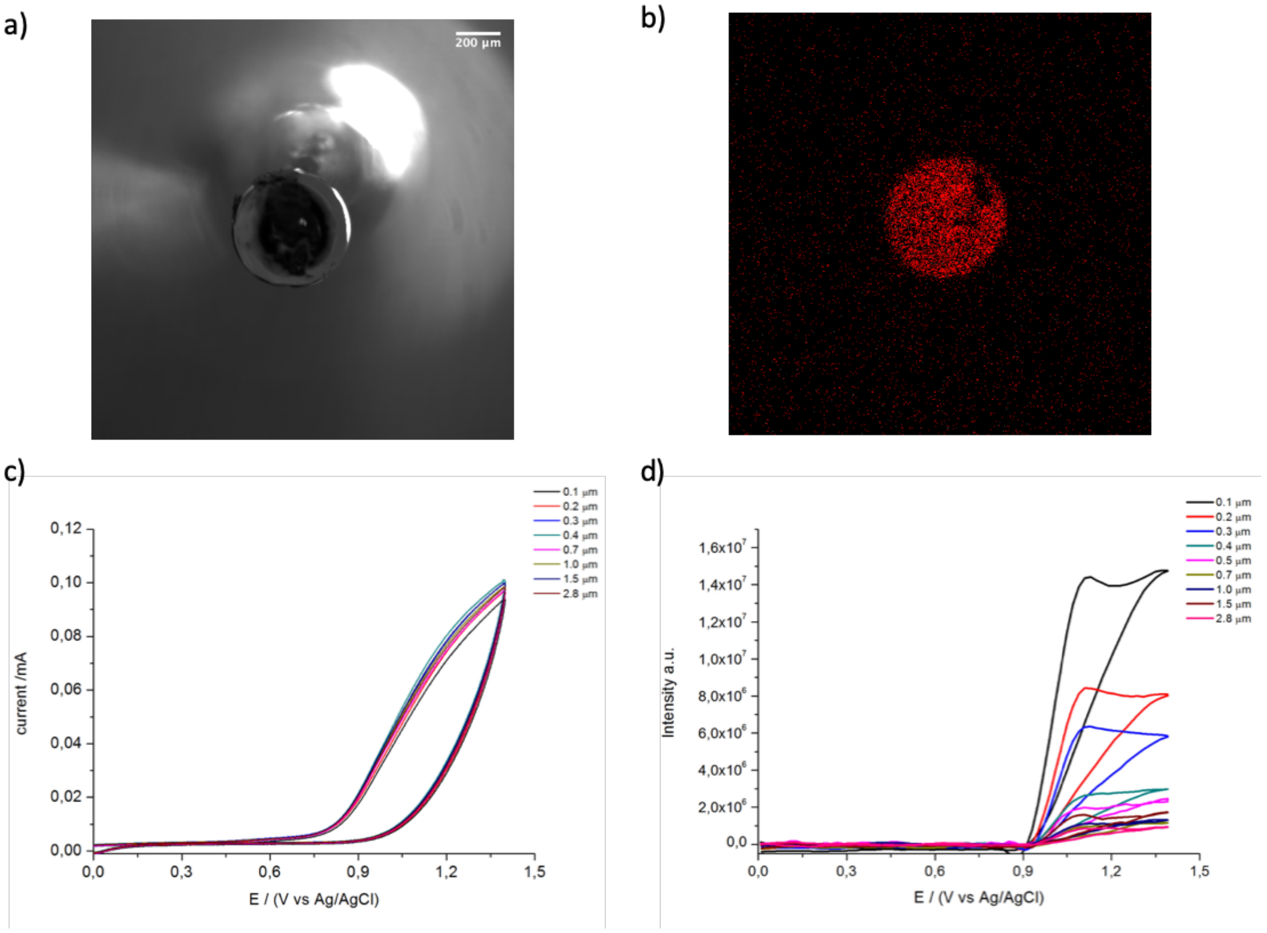

Supplementary Figure 8. Hemispherical Small electrode characterization. Images of **a)** optical front view of the electrode and **b)** ECL emission generated at 0.1 µm distance from the ITO surface modified with Ru complex at 1.4 V, Magnification 4x. Cyclic voltammograms **c)** currents and **d)** corresponding ECL signals at different distance (0.1 – 2.8 mm) from the ITO modified surface applying a potential of 0-1.4 V, scan rate 100 mV s^{-1}. All measurements were obtained in 0.2 M PB with TPrA 180 mM (pH 6.9), potential vs Ag/AgCl (3 M KCl). Scale bar, 200 µm.

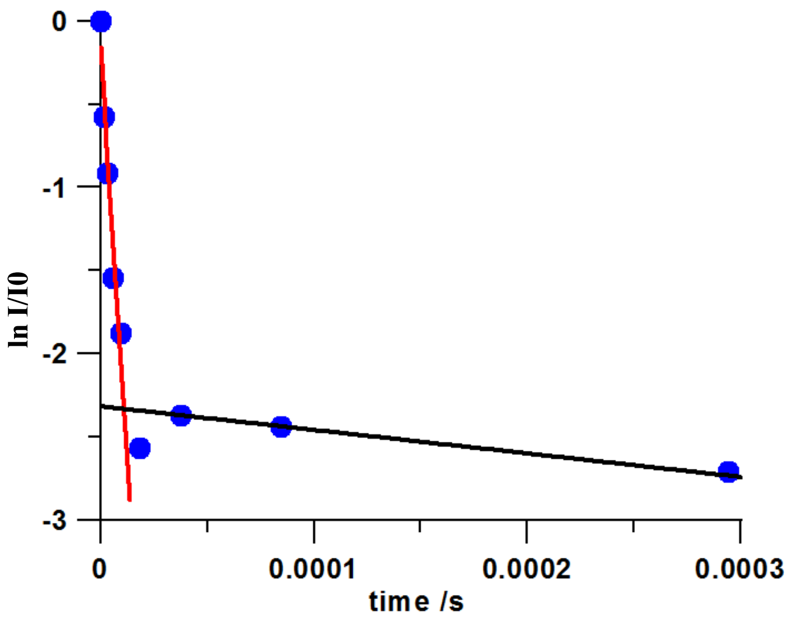

Supplementary Figure 9. Travel time calculation. Time resolved spatial map of ECL intensity calculated from Eq. (2), see main text. Data obtained with tip generation-substrate emission experiment using hemispherical small electrode (figure 2d, main text).

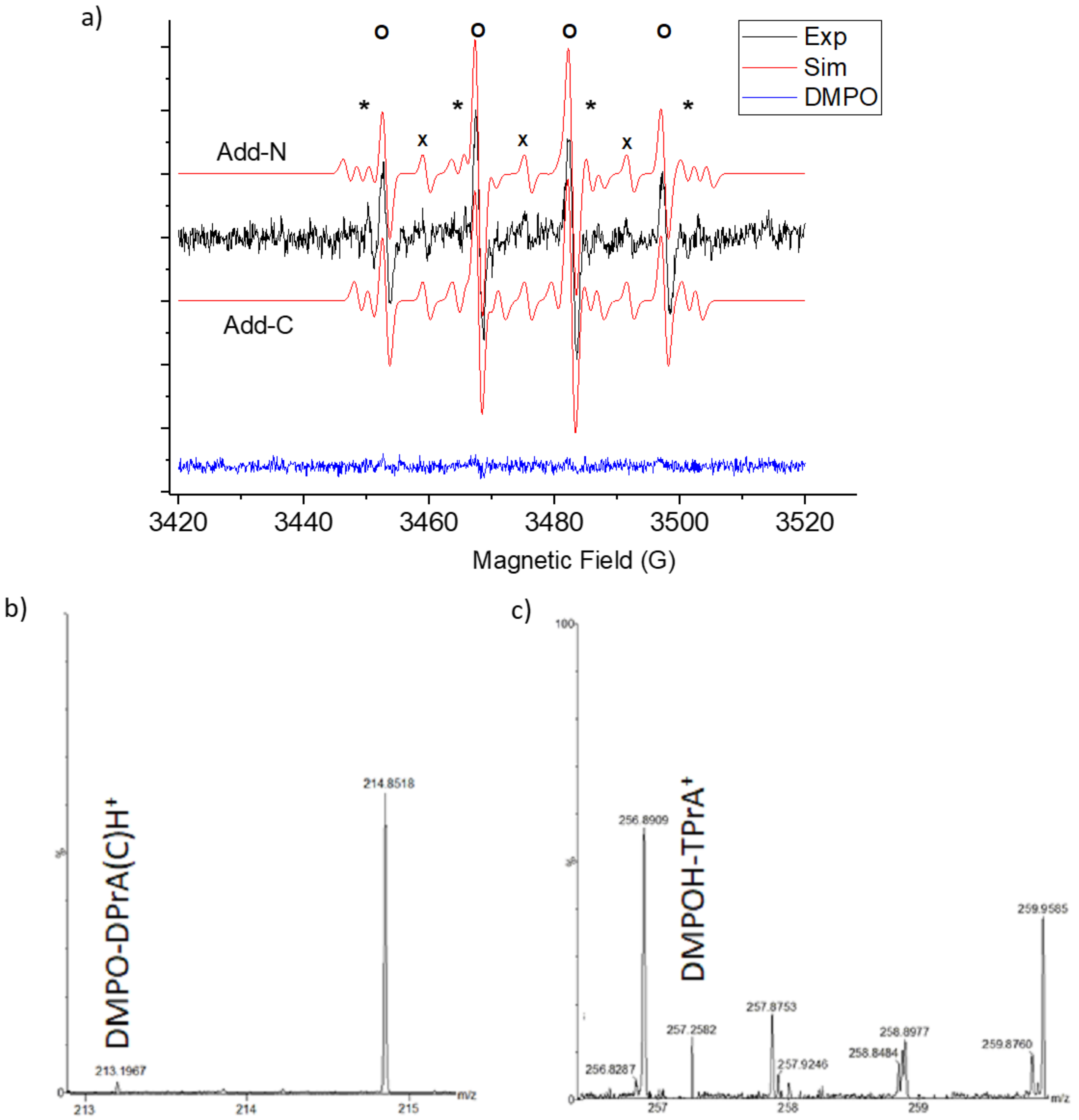

Supplementary Figure 10. Spin Trapping experiments and analysis of radical intermediates. Analysis of the adducts formed during 1h electrolysis at 1.4V vs Ag/AgCl 1 mL solution in Ar saturated solution of DMPO (=0.05 M)/TPrA (0.18 M) in formiate buffer at pH=6.9. **a)** EPR experimental spectrum (black line) and simulated (red lines) as sum of three adducts (accordingly with Supplementary table 2): DMPO-OH (**O**)+$RNO^{•}$ (**x**) + either an adduct compatible with an amine radical N-centered (Add-N) or a C-centered (Add-C) (*****). For reference, the spectrum of the solution of just the distilled DMPO exposed to air for 1 h is shown at the bottom (blue line). **b)** and **c)** Mass spectrum of a solution showing the formation of spin adducts DMPO-DPrA(C)H^{+} (m/z=213.1961) and DMPOH-TPrA (+H) $^{+}$ (m/z=257.2587); this label indicates the charged forms of DMPOH-TPrA(N)$^{+}$ and DMPOH-TPrA(C)-$H^{+,}$ with an extra proton (see supplementary methods 3 for details).

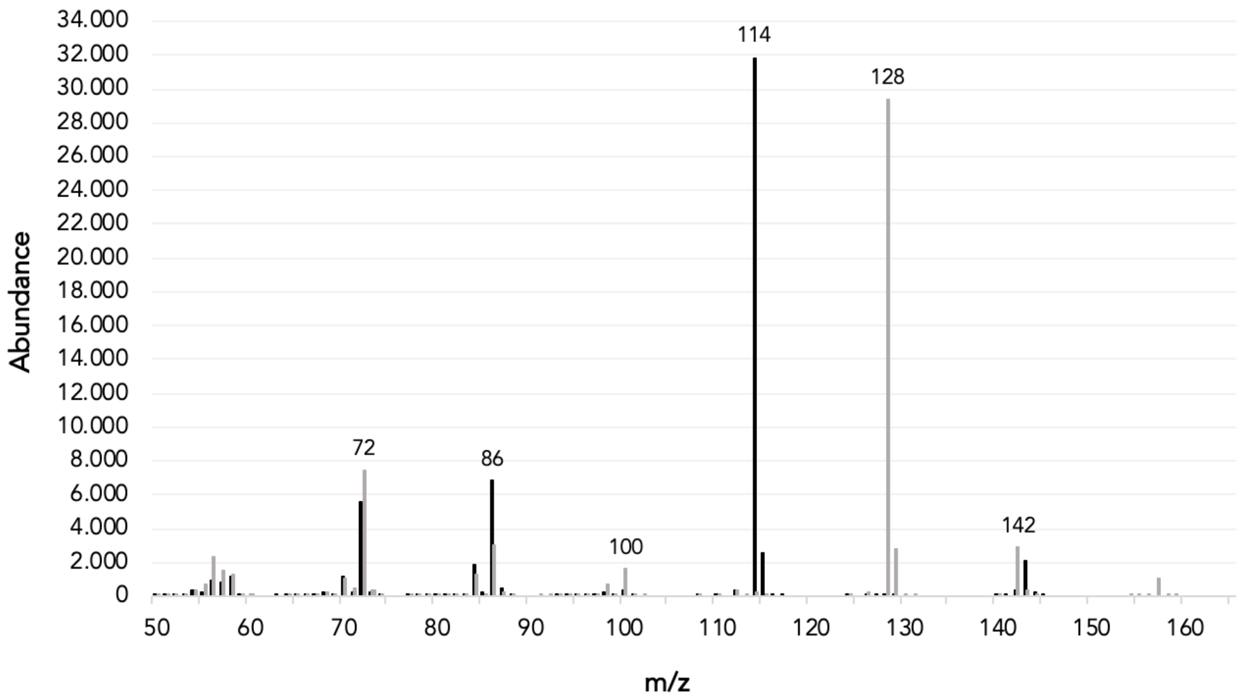

Supplementary Figure 11. Fragmentation patterns in the mass spectra of TPrA and DPIBA . Mass fragmentation (m/z) patterns of (black) TPrA and (grey) DPIBA. Notice that for the DPIBA the mass fragment with m/z 100 shows higher intensity indicating higher stability of the generated fragment.

a) $(CH_3CH_2CH_2)_3N\!:\ -1\ e^- \longrightarrow (CH_3CH_2CH_2)_3N^{+\bullet}$ TPrA(N)$^+$

b) $(CH_3CH_2CH_2)_3N^{+\bullet} \longrightarrow (CH_3CH_2CH_2)_2N\dot{C}HCH_2CH_3 + H^+$ TPrA(C)

c) $(CH_3CH_2CH_2)_3N\!:\ -1\ e^- \longrightarrow CH_3CH_2CH_2^+ + (CH_3CH_2CH_2)_2N^\bullet$ DPrA(N)

d) $(CH_3CH_2CH_2)_2N^\bullet \longrightarrow CH_3CH_2CH_2NH\dot{C}HCH_2CH_3$ DPrA(C)

e)

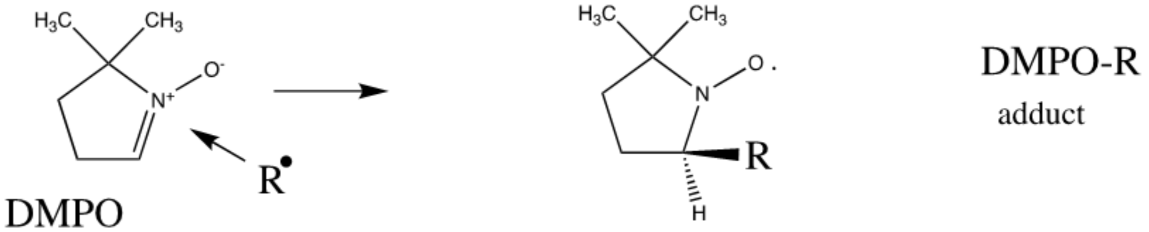

Supplementary Figure 12. Reactions involved in the spin trapping experiments. a-d) schematic reaction pathway for the oxidation of TPrA and the generation of TPrA and DPrA radicals. **e)** Spin trap 5,5-dimethyl-pyrroline N-oxide (DMPO) and formation of the spin-adducts by reaction with DMPO

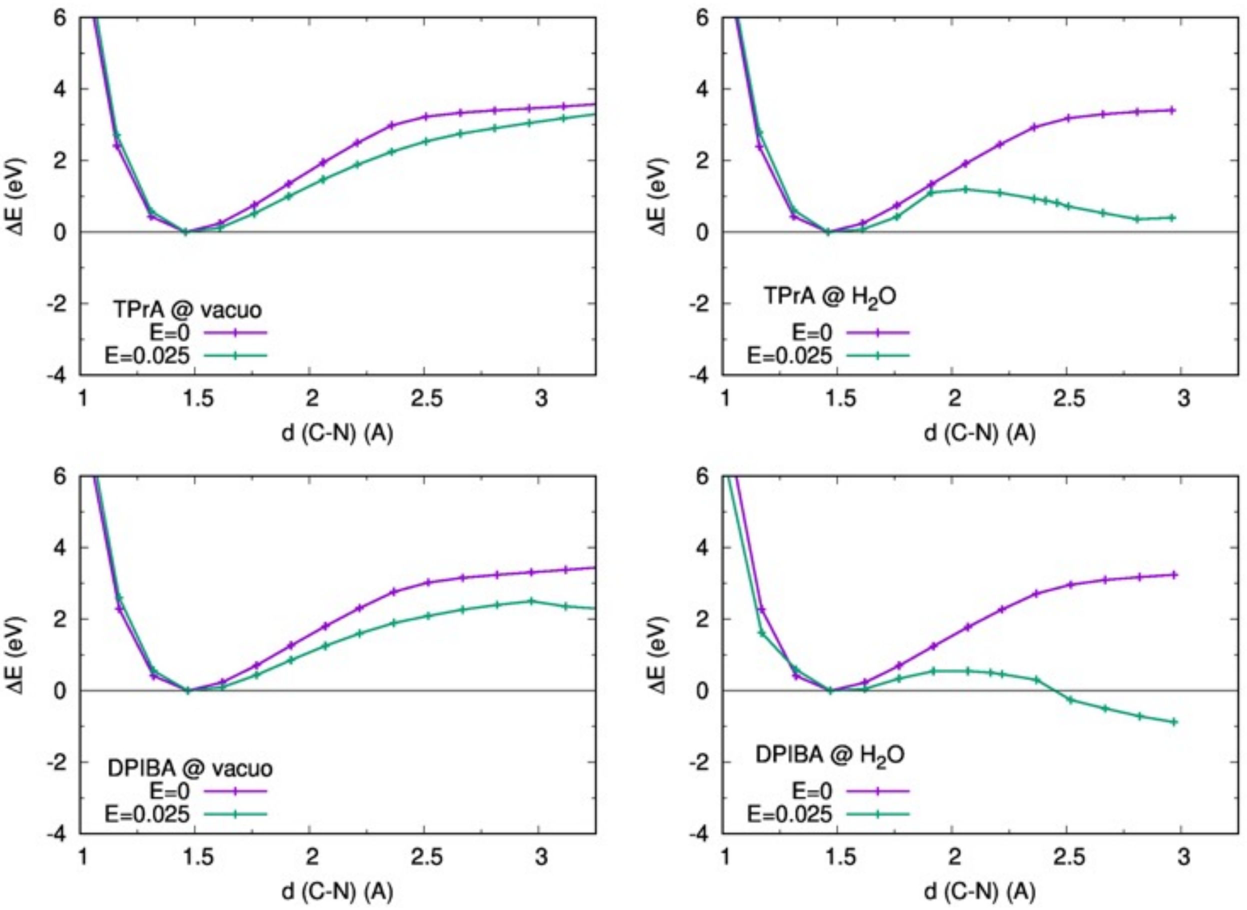

Supplementary Figure 13. **Potential Energy Curves (PES).** Computed PES (M062X/6-31+G* + IEFPCM) along C-N cleavage for TPrA **(top)** and DPIBA **(bottom)**, in vacuo **(left)** or in water **(right)**. The scan is computed in the absence (purple line) or in the presence of electric field (0.025 a.u green line).

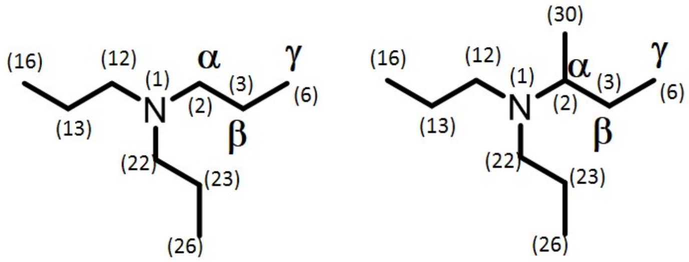

Supplementary Figure 14: TPrA **(left)** and DPIBA **(right)** structure with atom numbering in brackets.

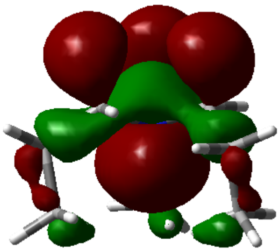

Supplementary Figure 15. The HOMO molecular orbital of TPrA.

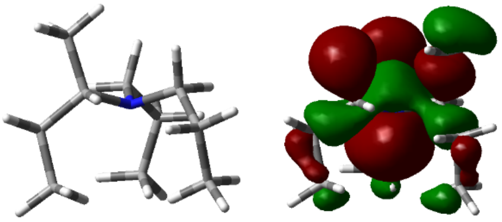

Supplementary Figure 16. The molecular structure **(left)** and HOMO molecular orbital **(right)** of DPIBA D1 conformer.

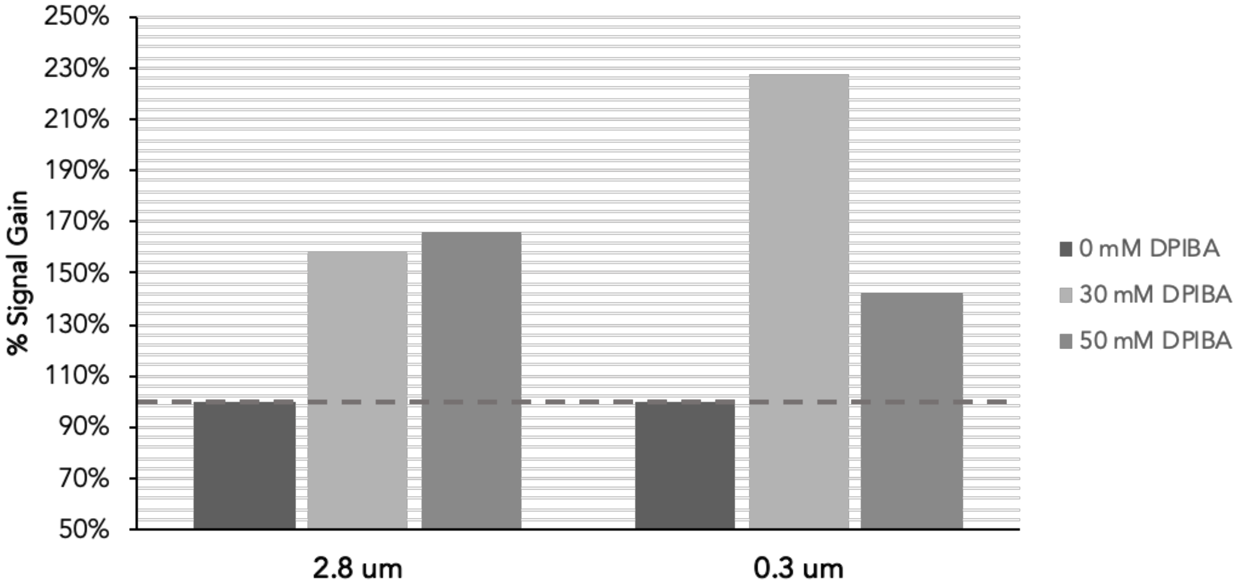

Supplementary Figure 17. ECL % Signal Gain. Signal gain % calculated from ECL intensities of 2.8 µm and 0.3 µm beads in TPrA 180 mM solution in PB 0.2 M (pH 6.9), with 30 mM and 50 mM of DPIBA as additive. Potential was applied for 4 s at 1.4 V, the scan rate was 100 mV s^{-1} and the magnification 100X.

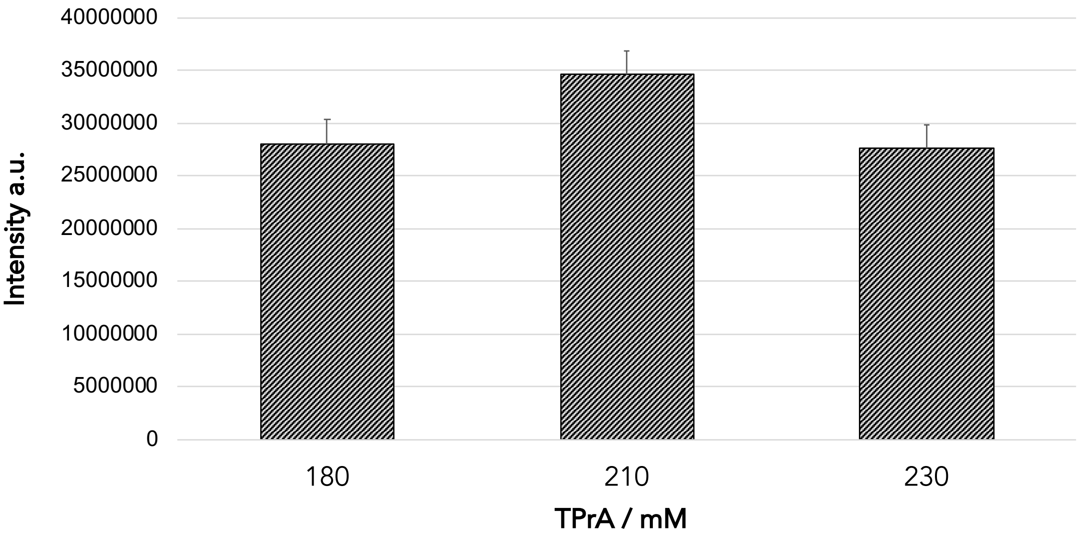

Supplementary Figure 18. Total amine concentration. ECL intensities from single bead experiments of 2.8 µm beads in TPrA solution in PB 0.2M (pH 6.9) with 180 mM, 210 mM and 230 mM of TPrA. Potential was applied for 4 s at 1.4 V. Magnification 100X. Error bar shows the standard deviation (n=10).

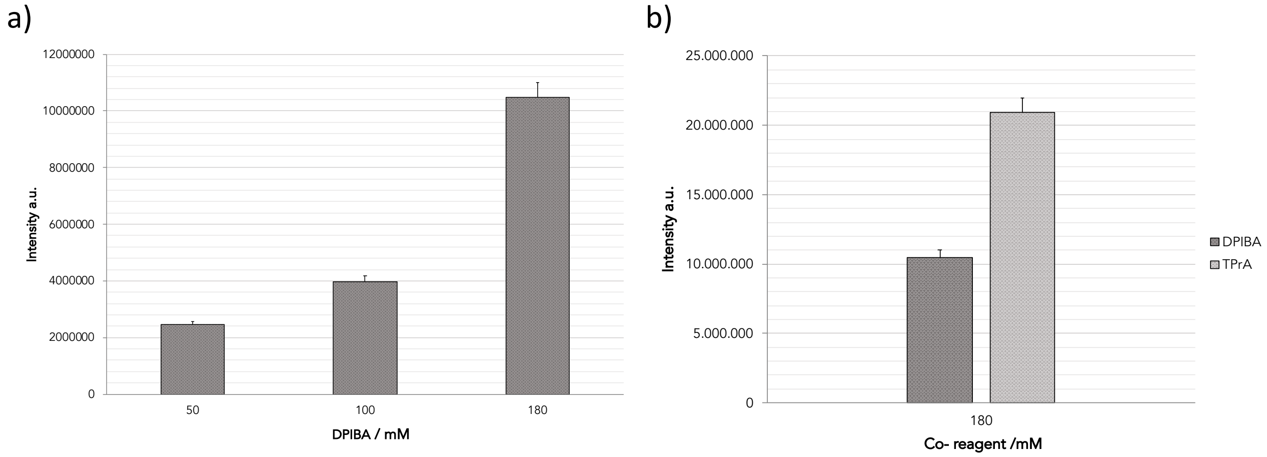

Supplementary Figure 19. ECL emission of DPIBA. Surface generation–bead emission experiment performed with **a)** 2.8 µm beads in 50, 100, and 180 mM DPIBA in 0.2 M PB (pH 6.9) and **b)** 2.8 µm beads in 180 mM DPIBA in 0.2 M PB (pH 6.9) and 180 mM TPrA in 0.2 M PB (pH 6.9). Potential was applied for 4 s at 1.4 V. Magnification, 100X. Error bar shows the standard deviation (n=10).

Supplementary Methods 1: surface generation-beads emission

Functionalization of beads with Ru (Ru@beads): Roche Diagnostics Elecsys® beads originating from the SAP assay (bottle M), diameter 2.8 µm; MyOne beads (ThermoFisher scientific): Dynabeads MyOne Streptavidin T1, diameter 1 µm; Bio-Adembeads Streptavidin (Ademtech): Bio-MasterBeads Streptavidin, diameter 500 nm and Bio-Adembeads Streptavidin, diameter 300 nm.

Roche Diagnostics Free Conjugate (antibody labelled with biotin and $Ru(bpy)_3^{2+}$) originating from the SAP assay (bottle R2).

For each bead dimension, the total surface area was constant of 7×10^9 µm^2. Indeed, the volume of bead solution was 133.9 (300 nm), 134.5 (500 nm), 265.6 (1 µm) and 6000.0 (2.8 µm) µl, respectively.

Each bead solution was poured in a 20 ml vial, collected with a magnet for 2 minutes, and afterwards the supernatant was discharged. Then, washing with 10 ml bead buffer was repeated 2 times for 5 minutes each. Beads were incubated with 18 ml of R2 (2 h at 37°C) by using a tube rotator. Separation was carried out with a magnet for 2 minutes and the supernatant was discharged. This whole procedure was repeated 5 times. Finally, beads were stored in the bead buffers from Roche Diagnostics SAP assay bottle M (2.8-1 µm) or Ademtech buffer (500-300 nm) in a total volume of 800 ml.

Ru quantification: The amount of Ru conjugated to beads (Ru@beads) was measured by inductively coupled plasma-mass spectrometry (ICP-MS).

Briefly, 500 µl of beads were dissolved in 358 ml of nitric acid (70%) and double distilled water for a final volume of 5 ml, and kept overnight at 80°C. After dissolution a clear solution was obtained.

The total amount of Ru, as ppb concentration, was normalized for the total surface area of each bead type, to obtain the density Ru µm^{-2}, as shown in the following table.

Supplementary table 1: Ru density (Ru µm^{-2}), Ru concentration ([Ru]/M) and Ru amount (n° Ru) calculated from Ru ppb concentration normalized for the total surface area of each bead type (0.3 nm, 0.5 nm, 1 µm, 2.8 µm).

Beads / µm	Total beads area	Area of 500 µl beads	[Ru] / ppb	[Ru] / M	n° Ru	Ru µm^{-2}
0.3	7.1E+09	4.4E+09	1.183	1.17E-08	3.52E+13	7969
0.5	7.1E+09	4.4E+09	1.262	1.25E-08	3.76E+13	8501
1	7.1E+09	4.4E+09	1.210	1.20E-08	3.60E+13	8151
2.8	7.1E+09	4.4E+09	0.652	6.45E-09	1.94E+13	4392

Turnover number (TON) and Turnover frequency (TOF)

TON and TOF are defined as:

$$TON = \frac{\int_0^t ECL_{Ru@bead} - \int_0^t ECL_{bead}}{n^\circ\ Ru@bead} \quad (1)$$

$$TOF = \frac{\int_0^t ECL_{Ru@bead} - \int_0^t ECL_{bead}}{n^\circ\ Ru@bead \times t} \quad (2)$$

ECL emission ($ECL_{Ru@bead}$) was quantified by integration of ECL images (.tiff), as obtained with the CCD camera in the surface generation-beads emission for a time (t) of 0.5 s. Software ImageJ was used to integrate the signals from ECL images with squares of 50×50px, 30×30px, 20×20px, and 20×20px for 2.8 µm, 1 µm, 0.5

µm, and 0.3 µm beads, respectively. Integrated ECL background (ECL_{bead}) was subtracted from the integrated ECL emission for each bead dimension. ECL_{bead} was measured with beads without Ru label.

ECL measurements using DPIBA as additive: To investigate the role of N-dipropyl isobutyl amine (DPIBA) (Roche Diagnostics)[1] we measured the ECL efficiency at a single bead level. Single bead images were recorded applying a constant potential of 1.4 V for 4 s with 8 s integration time with TPrA 180mM in 0.2 M PB (pH 6.9) solution containing DPIBA at different concentrations (30, 50 mM) or in DPIBA alone.
When we analyzed the % signal gain, where 0.2 M PB with 180 mM TPrA is used as reference, we observed an increase in the ECL emission when 30 and 50 mM of additive is used.
% Signal Gain was calculated using ECL intensity at TPrA 180 mM in 0.2 M PB without DPIBA as reference and is defined as follow:

$$\%\,Signal\ Gain = \frac{\text{ECL intensity at X mM DPIBA}}{\text{ECL intensity with 0 mM DPIBA}}\ \text{x}\ 100 \qquad (3)$$

Supplementary Methods 2: Tip generation-surface emission

Immobilization of Ru on an ITO Electrode: A clean, dried indium tin oxide (ITO) electrode with dimensions of 1.5 x 1.5 cm^2 was immersed in a 5% of $(CH_3O)_3Si(CH_2)_3NH_2$ toluene solution and kept in a desiccator for 24 h. During this process, $(CH_3O)_3$-$Si(CH_2)_3NH_2$ becomes immobilized by formation of ITO/O-$Si(CH_2)_3$ - NH_2 bonds. The electrode was then washed with EtOH and transferred into a 0.10 M 1-methylimidazole/HCl buffer solution (pH 7) containing 10 mM $[Ru(bpy)_2(4\text{-}COOH\text{-}bpy)]^{2+}$ and 10 mM EDC. After a 45 min incubation at 70 °C, the ITO electrode was washed thoroughly with EtOH and then water. With this reaction sequence, a layer of $[Ru(bpy)_2(4\text{-}COOH\text{-}bpy)]^{2+}$ was covalently attached to the aminosilane formed previously on the ITO, to produce ITO/O-$Si(CH_2)_3NH$-$[(CO\text{-}bpy)(bpy)_2Ru(II)]$. The substrate was subsequently immersed in a 0.10 M Tris/HCl buffer (pH 8) and maintained in the dark until further use.

Tip generation surface emission experimental procedure: Indium tin oxide (ITO) functionalized with $[Ru(bpy)_3]^{2+}$ was placed in a measuring cell together with a Pt counter electrode and an Ag/AgCl (3 M KCl) reference electrode, filled with TPrA 180 mM in 0.2 M PB (pH 6.9). Distance calibration of the Pt hemispherical electrode was done with TPrA 180 mM in 0.2M PB solution by slowly moving down (0.1 µm sec^{-1}) the tip while applying a constant potential of 1.2 V vs Ag/AgCl. A "CH Instruments CHI910B" apparatus controlled the tip potential and movement.

In this way, we could monitor the current as the tip approaches the ITO (see Supplementary Figure 5) until a current spike is observed, which represents the distance 0, i.e., when the tip is in contact with the underlying ITO support.

All the measurements were recorded moving the electrode from 0.1 µm to 2.8 µm distance applying a potential of 0-1.4 V in cyclic voltammetry and recording both currents and ECL signals (Supplementary Figure 6 c, d and 8 c, d).

As shown in Supplementary Figure 6c and 8c, when the electrode potential was scanned beyond 0.6 V we have an enhancement of the oxidation current due to the direct oxidation of coreactant TPrA,[2] which remains constant over all distances due to the large surface area. On the other hand, the initial ECL signal started following the oxidation of TPrA reaching a maximum at 1.2 V (Supplementary Figure 6d and 8d) following the "revisited" route.[3,4] ECL images recorded during this process (Supplementary Movie 1 and 2) confirmed that the ECL spot increases in intensity and in dimension as the potential grows. Supplementary Figure 6b taken at 1.4 V clearly shows a not homogeneous ECL emission generated with the large Pt electrode, but not observed with the small Pt electrode. This is a well-known behavior associated to hindrance of TPrA diffusion exerted by the large tip.[5]

Supplementary Methods 3: Spin Trapping experiments and analysis of radical intermediates

In order to unequivocally identify the radicals generated following the oxidation of TPrA we performed prolonged electrolysis and we used spin trapping for stabilization of the electrogenerated radicals (as it is schematized in Supplementary Figure 12). Spin trapping experiments allow to transform short-living radicals into long-living systems with (normally) paramagnetic character, therefore suitable to be characterized by Electron Paramagnetic Resonance (EPR) and mass spectrometry.

Spin-trapping experiments were conducted by adding the spin traps 5,5-dimethyl-pyrroline N-oxide (DMPO) (≥98%, for ESR, Sigma, 0.05 M before the electrolysis) to a solution of TPrA 180 mM, formic acid pH 6.9. The electrolysis was performed at 1.4 V (vs Ag/AgCl) for 1 h under Ar saturated atmosphere to reduce oxygen content. After electrolysis, the solution was analyzed by mass spectrometry and Electron Paramagnetic Resonance (EPR). According to literature [6,7] oxidation of TPrA can lead to the following radical products. In addition, the nitroxide group can lately reduce to give the analogous nitroxylamine (DMPOH) adduct.

EPR spectra: data in Supplementary Figure 10 were obtained with a Bruker Elexsys spectrometer operating at X-band and equipped with an ER4103 TM cavity; a flat cell (0.5 mL) was used for the sample. Acquisition was conducted at room temperature, and spectrometer parameters were microwave attenuation 10 dB (~20 mW), sweep width 10 mT, 1024 points, time constant 10.24 ms, modulation amplitude 0.12 mT, conversion time 40.96 ms, 5 scan/spectrum. To improve S/N, 20 acquisitions were summed.

The EPR spectrum (Supplementary Figure 10) confirms the formation of a spin adduct with an amine (asterisks) with typical values for the hyperfine coupling constants (hfcc) for nitroxyde N a_N=15.5-16 G and for the characteristic β-proton $a_{N-\beta}$ =18-23 G (see supplementary table 2 and spin-trap database[8,9]), but a difficult attribution to a trapping of either a N-centered (Add-N) or a C-centered (Add-N) adduct. Besides that, the spectrum shows the DMPO adduct with OH, and a normal nitroxide ($RNO^{\bullet}$), often present, and possibly formed from the decomposition of DMPO adducts.[10]

Supplementary Table 2. Fitting parameters of spectrum in Supplementary Figure 10. The tentative attribution of the C-centered radical or the N-centered radical spin adducts is conducted on the base of the Supplementary Figure 12 and Figure 3. The Greek labelling indicates the position with respect to the nitroxide N.

Adduct	a_N (G)	$a_{H-\beta}$ (G)	$a_{N-\beta}$ (G)	$a_{H-\gamma}$ (G)
DMPO-OH	14.95	14.67	/	/
Add-C	15.8	20.9	/	2.0
Add-N	17.4	19.4	2.0	/
$RNO^{\bullet}$	16.30	/	/	/

Mass spectrometry analysis: reported in Supplementary Figure 10 was performed by FFI (Fast Flow Injection) in a water/acetonitrile mixture (50/50 + 0.1% formic acid, 50 µl/min) on a Waters Xevo G2S QTof mass spectrometer (Milford, MA, USA) equipped with an electrospray (ESI) source. Capillary voltage was 1.5 kV in positive mode with a temperature of the source of 100 °C. Desolvation temperature was 350 °C while the gas flow were 10 L/h for the cone and 800 L/h for the desolvation.

Supplementary Table 3. Adduct obtained from the mass spectrometry analysis reported in Supplementary Figure 10 and Figure 2. For the structure schematization see Supplementary Figure 12.

Adduct	m/z
DMPOH-TPrA(N)$^+$	257.2587
DMPOH-TPrA(C)H$^+$	257.2587
DMPO-DPrA(N)H$^+$	214.2040
DMPO-DPrA(C)H$^+$	213.1961

The results of the mass spectra evidence the formation of species DMPO-DPrA(C), therefore, as reported for TPrA, the nitrogen radical cation undergoes a transposition to form a C-centered radical before to be trapped by DMPO.

For the primary radical of TPrA, instead, we observe a peak relative to ions with Z=1 of either species DMPO-TPrA(N)$^+$ and/or DMPO-TPrA(C)-H$^{+,}$ with an extra proton. This is not surprising, and often nitroxides in acid solution can undergo disproportion to hydroxylamine and oxammonium cation.[11]From the mass spectra, it is not possible to distinguish between the formation of adduct DMPOH-TPrA(C) and DMPOH-TPrA(N)$^+$, as the masses of the relative charged ions are the same

Therefore, mass spectra account for the formation of DMPO-DPrA(C), and likely of the precursor DMPO-DPrA(N), and for the formation of a C-centered or a N-centered radical adduct of TPrA with DMPO; for this adduct we have identified the reduced form, where the nitroxide turned into hydroxylamine. These last species have a larger signal that might be related to a larger concentration in the solution.

Supplementary Methods 4: Computational results

Starting geometry of TPrA and DPIBA: due to the flexible substituents of the TPrA molecule, as a first point the most stable conformer has been assessed as a function of the dihedral angle described by N-C_α-C_β-C_γ- of each alkyl chain, in neutral state (Supplementary Figure S14). A scan of molecular energy for varying dihedral angles (from -180 to 180°, scan step=15°) allowed to identify and optimize the most stable conformer, employed in subsequent calculations. The characteristics of the most interesting conformers identified from the scan are summarized in Supplementary Table 4. All of them have an N atom with sp3 hybridization and a pyramidal arrangement of C-N bonds. The most stable structure is labeled T1 (Supplementary Table 4, left), having all the alkyl chains similarly arranged with a dihedral angle of 60°. Other stable conformers have an appreciably higher relative energy with respect to the T1 structure, and consequently a significantly lower relative population, estimated by means of the Boltzmann distribution (< 0.3). Moreover, the different chain orientation does not determine significant differences regarding the C-N bond length, which is 1.46 Å for all conformers. Similar considerations hold true for the highest occupied orbital (HOMO, Supplementary Figure 15) energy, that is very close for all conformers, with differences below 0.05 eV. Hence in the subsequent discussion, only the most stable conformer was considered, namely T1.

Supplementary Table 4. Most relevant conformers of neutral TPrA, computed in vacuo. Dihedral angles of the alkyl chains, energy difference (ΔE) with respect to the most stable (Conformer T1, left), along with an estimate of Boltzmann population (Pop.Boltz.); HOMO energies, E(HOMO) and C-N bond length, d(C-N).

	T1	T2	T3	T4
	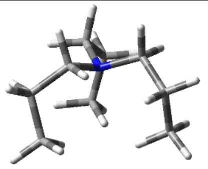	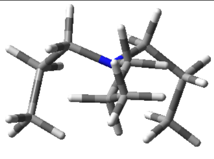	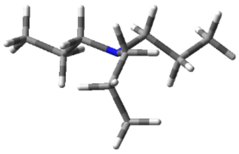	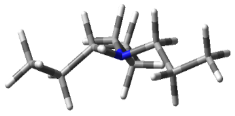
dihedral angles / °	60, 60, 60	60, 65, 180	65, 180, 180	180, 180, 180
ΔE / *kcal/mol* (Pop.Boltz)	0.00 (1.00)	0.68 (0.32)	1.33 (0.11)	1.82 (0.05)
E(HOMO) / *eV*	-7.42	-7.37	-7.39	-7.42
d (C-N) / Å	1.46 / 1.46 / 1.46	1.46 / 1.46 / 1.46	1.46 / 1.46 /1.46	1.46/ 1.46 /1.46

With regards to DPIBA, it has been assumed that the methylation does not significantly influence the alkyl chains orientation. Hence, only conformer D1, Supplementary Figure 16, has been considered. The effect of methylation has a minimal impact on this bond, since C-N involving the methylated $C_\alpha$is only slightly longer (1.47 Å) than the other C-N bonds (1.46 Å, similar as TPrA). The delocalization of HOMO density (Supplementary Figure S15) also on the methyl substituent destabilizes its energy only slightly with respect to TPrA (Energy of DPIBA = -7.25 eV to compare with TPrA = -7.42 eV, in vacuo)

C-N bond cleavage: the potential energy surfaces (PES) of dissociation for neutral TPrA and DPIBA were obtained by scanning the energy of the molecule along C-N bond stretching with a scan step = ± 0.15 Å. The scan was performed both in vacuo (Supplementary Figure 13 left, purple line) and in water (Supplementary Figure 13 right, purple line). This dissociation pathway is not competitive in the absence of an electric field owing to the large barrier. Therefore assuming an inner sphere electron transfer, we reevaluated the potential energy surface in the presence of an external electric field (***E***) applied along the C-N bond to be cleaved (directed towards the N atom) and with 0.025 a.u (~10^8 V/cm) of magnitude (Supplementary Figure 13, green line). For both TPrA and DPIBA, the resulting PESs flattens, with a marked tendency towards stabilizing the products in solvent, after overcoming a single barrier of ca. 1 eV for TPrA and ca. 0.6 eV for DPIBA.

To characterize the electronic structure evolution of the molecular fragments upon dissociation, the Mulliken spin densities are employed to identify unpaired radicals (Supplementary Table 3), along with the partial charges based on NBO (Natural Bond Orbitals) analysis;[12] in particular the total charge of the propyl (for TPrA) or isobutyl (for DPIBA) cleaved fragment is evaluated, as a sum of the NBO atomic partial charges of all the atoms belonging to the cleaved fragment (Supplementary Table 6). In the absence of the electric field (***E***=0), the C-N dissociation is covalent and leaves a localized unpaired electron on C(2) atom of the cleaved bond (Supplementary Table 5) without the formation of a neat charge on the fragment (Supplementary Table 6). On the contrary, in the presence of the field (***E*** = 0.025 a.u), the C-N dissociation acquires a ionic character with the formation of a carbocation (Supplementary Table 6); the ionic character is further confirmed by the absence of appreciable spin density on C(2) atom (Supplementary Table 5).

Supplementary Table 5. Spin densities on C atoms (see Supplementary Figure 13 for numbering) of cleaved propyl fragment for TPrA **(left)** and DPIBA **(right)** from calculations in H_2O without (***E*** =0) and with (***E*** = 0.025a.u) external electric field.

TPrA		
	E = 0	***E*** = 0.025
C(2)	1,06	0,03
C(3)	-0,12	-0,01
C(6)	0,01	0,00

DPIBA		
	E = 0	***E*** = 0.025
C(2)	-1,00	-0,01
C(3)	0,09	0,00
C(6)	0,00	0,01
C(30)	0,12	0,01

Supplementary Table 6. Cleaved propyl fragment final charge, computed from NBO partial charges, from calculations in H_2O without (***E*** =0) and with (***E*** = 0.025a.u) external electric field.

	E = 0	***E*** = 0.025
TPrA	0.0	+1.0
DPIBA	0.0	+1.0

Supplementary Methods 5: evaluating the effect of DPIBA on the performance of commercial immunoassays (Elecsys® assays)

All determinations were performed on a cobas® 8000 / **cobas e** 801 analyzer using the implemented assay protocols. Commercially available system reagents CleanCell M and PreClean II M, AssayTip/AssayCup trays, Elecsys® assay reagent kits and Elecsys calibrator sets were used (see Supplementary table 7). All calibrator sets were reconstituted as indicated in the product insert. ProCell II M was replaced by 0.2 M PB (pH 6.9) with 180 mM TPrA and 0.1% polidocanol, which were supplemented with 0 mM (reference), 30 mM, 50 mM and 100 mM DPIBA. Conductivity (25 mS cm^{-1}) and pH (6.9) were similar among these buffers. The obtained ECL signals [counts] with 30 mM and 50 mM DPIBA were normalized against the signals obtained with the reference buffer lacking DPIBA (signal gain [%]).

Supplementary table 7. Material numbers for Elecsys assay reagent kits and calibrator sets.

Elecsys assay reagent kit		Elecsys calibrator set		test principle
material number	name	material number	name	
07028091190	TSH	04738551190	TSH CalSet	sandwich
07028075190	Troponin T hs	07401671190	CalSet Troponin T hs	sandwich
07027273190	Ferritin	03737586190	Ferritin CalSet	sandwich
07028024190	Toxo IgM	included in reagent kit		µ-capture
07026773190	Anti-HAV IgM	included in reagent kit		µ-capture

Supplementary Bibliography:

1. Josel, H.-P., Andres, H., Windfuhr, M., Larbolette, O. & Quint, S. Branched-chain amines in electrochemiluminescence detection. WO 2017/153574 A1 (2017).

2. Miao, W., Choi, J. P. & Bard, A. J. Electrogenerated chemiluminescence 69: The Tris(2,2'-bipyridine)ruthenium(II), ($Ru(bpy)_3^{2+}$)/tri-*n*-propylamine (TPrA) system revisited - A new route involving $TPrA^{\bullet+}$ cation radicals. *J. Am. Chem. Soc.* **124**, 14478–14485 (2002).

3. Voci, S. *et al.* Surface-confined Electrochemiluminescence Microscopy of Cell Membranes. *J. Am. Chem. Soc.* **140**, 14753–14760 (2018).

4. Valenti, G., Fiorani, A., Li, H., Sojic, N. & Paolucci, F. Essential Role of Electrode Materials in Electrochemiluminescence Applications. *ChemElectroChem* **3**, 1990–1997 (2016).

5. Amemiya, S., Bard, A. J., Fan, F. F., Mirkin, M. V & Unwin, P. R. Scanning Electrochemical Microscopy. *Annu. Rev. Anal. Chem.* **1**, 95–131 (2008).

6. Das, S. & Sonntag, C. Von. The Oxidation of Trimethylamine by OH Radicals in Aqueous Solution , as Studied by Pulse Radiolysis , ESR , and Product Analysis . The Reactions of the Alkylamine Radical Cation , the Aminoalkyl Radical , and the Protonated Aminoalkyl Radical. *Naturforsch.* **41b**, 505.513 (1986).

7. Adenier, A., Chehimi, M. M., Gallardo, I. & Pinson, J. Electrochemical Oxidation of Aliphatic Amines and Their Attachment to Carbon and Metal Surfaces. *Langmuir* **20**, 8243–8253 (2004).

8. https://tools.niehs.nih.gov/stdb/index.cfm/spintrap/.

9. Migita, C. T. & Migita, K. Spin Trapping of the Nitrogen-centered Radicals. Characterization of the DMPO / DEPMPO Spin Adducts. *Chem. Lett.* **32**, 466–467 (2003).

10. Zerbi, G. et al. Graphite particles induce ROS formation in cell free systems and human cells. *Nanoscale* **9**, 13640–13650 (2017).

11. Sen, V. D. & Golubev, V. A. Kinetics and mechanism for acid-catalyzed disproportionation of 2,2,6,6-tetramethylpiperidine-1-oxyl. *J. Phys. Org. Chem.* **22**, 138–143 (2009).

12. E. D. Glendening, A. E. Reed, J. E. Carpenter, F. W. *NBO Version 3.1*.
