# Supplementary material for: Insights into the mechanism of coreactant electrochemiluminescence facilitating enhanced bioanalytical performance
Source: Nat Commun. 2020 May 29;11:2668. doi: 10.1038/s41467-020-16476-2 (PMC7260178; doi:10.1038/s41467-020-16476-2)
Supplement: Supplementary file 3 — Description of Additional Supplementary Files [file 41467_2020_16476_MOESM3_ESM.docx]

**Description of Supplementary Files**

**File Name: Supplementary Movie 1**

**Description:** ECL imaging of large electrode taken at 0.1 µm distance from the Ru@ITO emitting surface during a CV scanning in TPrA 180mM in 0.2M Phosphate Buffer (PB, pH 6.9). Potential 0-1.4 V vs. Ag/AgCl (3 M KCl), scan rate 100 mV s-1, exposure time 200 ms, and magnification 4x.

**File Name: Supplementary Movie 2**

**Description:** ECL imaging of small electrode taken at 0.1 µm distance from the Ru@ITO emitting surface during a CV scanning in TPrA 180mM in 0.2M Phosphate Buffer (PB, pH 6.9). Potential 0-1.4 V vs. Ag/AgCl (3 M KCl), scan rate 100 mV s-1, exposure time: 200 ms, and magnification 4x.
